# The impact of a community-based intervention on weight, weight-related behaviours and health-related quality of life in primary school children in Victoria, Australia, according to socio-economic position

**DOI:** 10.1186/s12889-021-12150-4

**Published:** 2021-11-27

**Authors:** Jane Jacobs, Claudia Strugnell, Steven Allender, Liliana Orellana, Kathryn Backholer, Kristy A. Bolton, Penny Fraser, Ha Le, Andrew Dwight Brown, Melanie Nichols

**Affiliations:** 1 Global Obesity Centre (GLOBE)Institute for Health Transformation, Faculty of Health, Deakin University, Geelong, Australia; 2 Biostatistics Unit, Faculty of Health, Deakin University, Geelong, Australia; 3 Deakin Health Economics, Institute for Health Transformation, Faculty of Health, Deakin University, Burwood, Australia

## Abstract

**Background:**

Approximately a quarter of Australian children are classified as overweight or obese. In high-income countries, childhood obesity follows a socio-economic gradient, with greater prevalence amongst the most socio-economically disadvantaged children. Community-based interventions (CBI), particularly those using a systems approach, have been shown to be effective on weight and weight-related behaviours. They are also thought to have an equitable impacts, however there is limited evidence of their effectiveness in achieving this goal.

**Methods:**

Secondary analysis was conducted on data collected from primary school children (aged 6–13 years) residing in ten communities (five intervention, five control) involved in the Whole of Systems Trial of Prevention Strategies for Childhood Obesity (WHO STOPS) cluster randomised trial in Victoria, Australia. Outcomes included Body Mass Index z-score (BMI-z) derived from measured height and weight, self-reported physical activity and dietary behaviours and health related quality of life (HRQoL). Repeat cross-sectional data from 2015 (*n* = 1790) and 2019 (*n* = 2137) were analysed, stratified by high or low socio-economic position (SEP). Multilevel linear models and generalised estimating equations were fitted to assess whether SEP modified the intervention effect on the outcomes.

**Results:**

There were no overall changes in BMI-z for either SEP strata. For behavioural outcomes, the intervention resulted in a 22.5% (95% CI 5.1, 39.9) point greater improvement in high-SEP compared to low-SEP intervention schools for meeting physical activity guidelines. There were also positive dietary intervention effects for high SEP students, reducing takeaway and packaged snack consumption, although there was no significant difference in effect between high and low SEP students. There were positive intervention effects for HRQoL, whereby scores declined in control communities with no change in intervention communities, and this did not differ by SEP.

**Conclusion:**

The WHO STOPS intervention had differential effects on several weight-related behaviours according to SEP, including physical activity. Similar impacts on HRQoL outcomes were found between high and low SEP groups. Importantly, the trial evaluation was not powered to detect subgroup differences. Future evaluations of CBIs should be designed with an equity lens, to understand if and how these types of interventions can benefit all community members, regardless of their social and economic resources.

**Supplementary Information:**

The online version contains supplementary material available at 10.1186/s12889-021-12150-4.

## Background

A quarter of children in high-income countries have overweight or obesity [1] following a clear socio-economic gradient with prevalence increasing when socio-economic position (SEP) decreases [2]. There is evidence that childhood obesity is plateauing overall in high-income countries [3] though these overall trends may be obscuring widening inequalities between children from high and low SEP backgrounds [4–6]. Further, weight-related behaviours, such as physical activity (PA) and diet, also vary according to SEP [4, 7, 8].

Childhood obesity, and socioeconomic inequalities in childhood obesity distribution, arise from complex interactions between individual, behavioural, environmental and social factors [9]. For example, weight-related behaviours in children may be driven by a combination of individual factors, such as family income and parental education levels [10], and environmental and social factors, such as access and affordability of healthy food, and opportunities for safe PA and active transport [11].

The World Health Organization (WHO) recommends that for effective and sustainable childhood obesity prevention, community-based interventions (CBIs) are delivered alongside population-wide healthy eating and active living policies and structural change [12]. CBIs are designed and delivered at the community level and driven by community members. Specific actions are devised that are specific to the community environment and implemented across multiple settings (e.g. schools, child care centres, neighbourhoods, sporting clubs) [12]. Systems thinking provides the tools to consider communities holistically, emphasising connections between parts of the community and wider system in planning interventions [13]. Applying a systems thinking approach as a way to develop actions for CBIs has received particular attention as a potential way to address health inequities [14–16], however evaluation of systems-based CBIs from an equity perspective is lacking [16].

Further to this, while the evidence is accumulating that CBIs can have equitable impacts on weight-related outcomes in obesity prevention studies [17], differential impacts according to SEP of CBIs on weight-related behaviours (e.g. PA and diet) and health-related quality of life (HRQoL) outcomes are often not evaluated. A systematic review [17] examining the impact of CBIs on a variety of health outcomes included five studies focussed on childhood obesity. Four of the five studies found the interventions to be equally or more effective in low compared to high SEP participants in preventing weight gain, with only one [18] study examining weight-related behaviours (PA) by SEP.

As obesity prevention interventions generally aim to improve PA and dietary behaviours, it is important to also evaluate the differential effect of interventions on these outcomes by SEP. Improvements in these behaviours may not always translate directly to weight outcomes, however could indicate that the intervention is positively impacting on intermediate variables (mediators) in the pathway between intervention and weight-related outcomes [16].

The current study presents a secondary analysis of data from the Whole of Systems Trial of Prevention Strategies for Childhood Obesity (WHOSTOPS Childhood Obesity), a four-year systems-based CBI involving ten communities in Victoria, Australia [19]. The trial found a significant within-group reduction in overweight/obesity prevalence in intervention communities between 2015 and 2017, followed by a significant increase between 2017 and 2019, while remaining constant in control communities across the four years [20]. Positive intervention effects were found for takeaway food consumption, HRQoL scores and nutrient poor snack consumption (boys) and water consumption (girls) [20]. The relatively long four-year follow-up period was one proposed explanation for the weight status outcomes, with reduced intensity of the intervention effort in the 2017–2019 time period. Comparable CBIs that have demonstrated reductions in overweight/obesity had one to two year follow-up periods [21–23], similar to the initial two-year results of WHO STOPS [20]. Investigation of the WHO STOPS results, according to SEP, may give further insight into the trial outcomes.

This study aimed to test the hypothesis that a CBI applying techniques from system science to prevent childhood obesity has differential impacts on weight status, weight-related behaviours and health-related quality of life according to school-level SEP in Victoria, Australia.

## Methods

This design, conduct and reporting of this study follow the recommendations of the Consolidated Standards of Reporting Trials (CONSORT): extension for Cluster Randomised Control Trials guidelines [24].

### Study design

This study is a secondary analysis of the WHO STOPS Childhood Obesity trial. Detailed study methods have been published elsewhere [19]. WHO STOPS employed a systems-based CBI approach to childhood obesity prevention and was implemented across ten communities in a regional area of Victoria, Australia. Ten communities were randomised (1:1) in 2015 into five intervention and five control communities. The original design was a stepped wedge design with control communities planned to enter the intervention in 2017. However, due to unplanned staff turnover in partner organisations, shifting priorities and a need for local partners to respond to natural disasters (e.g., bushfires), the intervention was delayed in control communities until 2019. Therefore, the communities remained intervention or control for the four-year period (2015–2019).

### Intervention

The intervention included five phases [19]. The first involved collection of the baseline height and weight, weight-related behaviours and health-related quality of life from primary school students in the ten communities. The second phase included working with leaders in the five intervention communities to identify the childhood obesity drivers in their community. These drivers were used develop a causal loop diagram (systems map) [25] to highlight the interconnections between these drivers. In the third phase, engagement of further community representatives was undertaken, particularly community members who could impact food and PA environments and choices. This group collaborated to identify intervention actions that could be undertaken in their community, based on the systems map and presentation of case studies of previously successful actions, known as the fourth phase. The final phase included monitoring of children’s weight status weight-related behaviours every two years in all ten communities, and ongoing review and updates to the systems map and actions conducted in the five intervention communities.

#### Community actions

The specific actions, number of strategies and intensity of intervention varied in each community depending on their unique circumstances, such as capacity, resources, community priorities and engagement. Examples of healthy eating initiatives included healthier food provision at school canteens, child-care centres and community playgroups, reduction or removal of sugar sweetened beverages (SSB) in health services and community centres, and increasing the availability of water and healthy food options at community events. Physical activity initiatives included providing designated drop off zones around schools to encourage children to walk the last 800 m, construction of a new footpath enabling improved active transport to school, walking school buses to primary schools, establishment of running groups and public running events in the community and teaching bike safety within schools [26].

### School recruitment and study procedures

At each monitoring wave (2015, 2017 and 2019) all primary schools (Government, Independent and Catholic) across the 10 communities were invited to participate. Students were enrolled into the study unless an opt-out form signed by their parents or guardians was returned to the school or verbal assent was not given by the student at the time of measurement. Students were also able to participate in as much (e.g. all measurements) or as little (e.g. only survey) as desired. In 2015, students in Catholic schools participated under an ‘opt-in’ approach, requiring active parental consent. Due to research demonstrating opt-in approaches underestimate population-level overweight/obesity by 5 percentage points [27], students from these schools were excluded in 2015 analyses.

School participation rates were 72.7% (40/55), and 62.9% (44/70) in 2015 and 2019 respectively. In 2015, the participation rate was 79.6% (1792/2251) of eligible students within participating schools, and in 2019 it was 78.6% (2137/2720).

Height and weight data were measured of Grade 2 (aged approx. 7–8 years), Grade 4 (aged approx. 9–10 years) and Grade 6 (aged approx. 11–12 years) students; and weight-related behaviours and HRQoL of Grade 4 and 6 students were collected by self-report questionnaire [28]. Measures were taken in 2015, 2017 and 2019 (only data from 2015 and 2019 are included in this study). All data were collected at the school, during school time.

### Variables

#### Socio-economic position (SEP)

The Index of Community Socio-Educational Advantage (ICSEA) scores for each school were used as an indicator of SEP. Scores were obtained from the Australian Curriculum, Assessment and Reporting Authority ‘My School’ website [29]. Scores are based on a combination of individual student factors (parental occupation and parental income) and school-based factors (geographic location of the school and proportion of Indigenous students). This score gives an overall indication of the school community’s relative SEP, with a national median value of 1000. ICSEA scores were dichotomised, categorising school as high (ICSEA ≥1000) or low (ICSEA < 1000) SEP [29].

*Weight status:* Height and weight of Grade 2, 4 and 6 students were measured by trained staff according to a standardised protocol [28]. Weight was measured to the nearest 0.05 kg and height to the nearest 0.1 cm. Measurements were taken twice and if they differed by more than 0.5 cm or 0.1 kg, a third measurement was taken. The average of height and weight measures was used to calculate body mass index z-scores (BMI-z) based on the WHO Child Growth Reference [30]. Weight status was categorised using the WHO recommended cut-offs; overweight: + 1 < BMI-z < + 2, obese: BMI-z > + 2 [30].

#### Demographics

Data on gender and age were collected for Year 2 students. Year 4 and 6 students were guided through questionnaires by trained staff, using tablet computers to record their answers. Students reported gender, date of birth, language usually spoken at home, Aboriginal and/or Torres Strait Islander background, residential postcode, and country of birth.

#### Physical activity behaviours

The Core Indicators and Measures of Youth Health – Physical Activity & Sedentary Behaviour Module questionnaire [31] was used to assess PA and sedentary behaviour and active transport. During the study time, the Australian Physical Activity guidelines were replaced with the 24 h movement guidelines [32], however the recommendations for PA and sedentary time did not change. Physical activity guideline adherence was defined as children participating in at least 60 min of moderate to vigorous physical activity (MVPA) on 5 or more days a week (yes/no). Adherence to the sedentary behaviour guidelines was defined as 2 h or less of sedentary screen time outside of school hours on 5 or more days a week (yes/no). Active transport was dichotomized as using an active form of transport to or from school (walking, cycling, public bus or other active) or no active form of transport (car, school bus or other inactive).

#### Dietary Behaviours

The Simple Dietary Questionnaire [33], which is based on the Australian Dietary Guidelines [34], was used to assess dietary behaviours. Included items were weekly consumption of fruit and vegetables, SSB, takeaway food, packaged snacks and water. Children were classified as meeting, or not meeting fruit and vegetable intake guidelines (fruit ≥2 serves a day; vegetables ≥5 serves a day, or ≥ 5.5 serves for boys 12-18 years [34]). SSB consumption and packaged snacks were dichotomised into ‘one or less per day’ or ‘more than one per day’, takeaway meals were dichotomised as ‘one or less per week’ or ‘two or more per week’. Water was classified as ‘less than five glasses (250ml) per day’ or ‘five or more glasses per day’ based on the recommendations for children 9–13 years of age [35].

#### Health-related quality of life

Health related quality of life was assessed using the 23-item Paediatric Quality of Life Inventory 4.0 (PedsQL) [36]. The PedsQL comprises 4 domains: physical functioning (8 items), emotional functioning (5 items), social functioning (5 items) and school functioning (5 items). From these domains, three scores are calculated; physical functioning, psychosocial (emotional, social and school functioning) and global (combining all domains) [36]. Scores range on a scale of 0–100, with higher scores representing higher HRQoL. A change of 4.5 points in the PedsQL global score is considered the minimum clinically important difference [36].

### Statistical analysis

Repeat cross-sectional data from 2015 and 2019 were included in the analysis. Analysis was conducted on an intention-to-treat basis. All children in Grades 2, 4 and 6 who had valid height and weight measurements were included for the weight status outcomes, and Grade 4 and 6 students with valid questionnaire responses were included for the weight-related behaviours and HRQoL surveys. A *p*-value of < 0.05 was considered significant in all analyses.

All analyses are presented stratified by SEP (low/high). Linear mixed models were used to measure the intervention effect on continuous outcomes (BMI-z, HRQoL scores), with school as a random effect to adjust for clustering. Generalised estimating equations were used for binary outcomes. All models included trial arm (intervention/control), timepoint, SEP (low/high) and the two and three-way interaction terms between group, timepoint and SEP. Models also included gender, age and school type (Government, Independent or Catholic) to adjust for potential confounding. For each outcome, we report a) the estimate mean (continuous outcomes) or prevalence (binary outcomes) by timepoint, trial group and SEP level; b) the estimated change between 2019 and 2015 within trial group and SEP level; c) the estimated intervention effect within SEP level; and d) the estimated modification of effect by SEP, alongside with the correspondent 95% confidence intervals (CI). All analyses were conducted in Stata SE version 15.0 [37].

Supplementary analysis, using the same approach outlined above, was undertaken including a four way interaction term between group, timepoint, SEP and gender as gender differences had been found for a number of variables in the overall WHO STOPS analysis [20].

## Results

There were 1790 students included in the 2015 sample and 2137 students in the 2019 sample. Student demographics in 2015 and 2019, stratified by intervention condition (intervention/control) and SEP (high/low), are shown in Table 1. Low SEP students dominated the sample in both control (77%) and intervention (75%) groups in 2015, but were more evenly spread in 2019, with 56% low SEP in control, 52% low SEP in intervention.

**Table 1:** 2015 and 2019 demographics stratified by intervention group and SEP

	Intervention		Control		Total
	Low SEP	High SEP	Low SEP	High SEP	
**2015**					
n (% of total)	726 (40.6%)	244 (13.6%)	635 (35.5%)	185 (10.3%)	1790
Age mean (sd)	9.7 (1.7)	9.6 (1.7)	9.8 (1.6)	10.0 (1.6)	9.7 (1.7)
Female (%)	48.5%	48.4%	51.0%	48.9%	48.9%
**2019**					
n (% of total)	456 (21.3%)	422 (19.7%)	708 (33.1%)	551 (25.8%)	2137
Age mean (sd)	9.8 (1.7)	9.9 (1.7)	9.8 (1.7)	9.9 (1.7)	9.9 (1.7)
Female (%)	45.8%	50.2%	45.3%	46.8%	46.8%

SEP: Socio-Economic Position; sd: standard deviation.

### 2015 Results

The 2015 results (Table 2) show no differences in weight status. For behaviours, within control communities, there was a higher prevalence of students meeting PA and fruit guidelines, having less than one takeaway per week, and one or less packaged snack and SSB per day among students attending high SEP schools (herein referred to as ‘high SEP students’) compared to students attending low SEP schools (herein referred to as ‘low SEP students’). In intervention communities, there was a lower level of SSB consumption in high SEP compared to low SEP students.

**Table 2:** Anthropometric, weight-related behavioural and health-related quality of life outcomes, stratified by SEP

	Intervention				Control				Change 2019–2015		Intervention effect (difference in change)	Effect modification by SEP (difference in intervention effect between SEP levels)
	2015		2019		2015		2019		Intervention	Control	Int - Control	High - low SEP
	n	Estimate(95% CI)	n	Estimate(95% CI)	n	Estimate(95% CI)	n	Estimate(95% CI)	Estimate(95% CI)	Estimate(95% CI)	Estimate(95% CI)	Estimate(95% CI)
**Anthropometry**												
*BMI z score (mean)*												
Low SEP	724	0.67 (0.57, 0.76)	452	0.80 (0.68, 0.92)	630	0.61 (0.51, 0.72)	692	0.72 (0.61, 0.82)	0.14 (0, 0.28)	0.10 (−0.02, 0.23)	0.03 (−0.15, 0.22)	0.23 (− 0.11, 0.56)
High SEP	242	0.68 (0.53, 0.84)	416	0.74 (0.60, 0.87)	185	0.68 (0.50, 0.86)	536	0.47 (0.33, 0.61)	0.05 (−0.14, 0.25)	−0.21 (− 0.42, 0.01)	0.26 (− 0.02, 0.54)	
O*verweight or obese (prevalence)*												
Low SEP	724	36.0 (32.0, 40.0)	452	41.4 (36.4, 46.5)	630	35.1 (30.8, 39.4)	692	38.2 (34.1, 42.4)	5.5 (−0.3, 11.3)	3.2 (− 2.1, 8.4)	2.3 (− 5.5, 10.2)	6.1 (− 7.9, 20.1)
High SEP	242	36.7 (30.2, 43.2)	416	40.4 (34.5, 46.2)	185	35.1 (27.8, 42.5)	536	30.4 (25.1, 35.7)	3.7 (−4.6, 12.0)	− 4.7 (−13.6, 4.2)	8.4 (− 3.2, 20.1)	
**Weight-related behaviours** (prevalence)												
*Meets physical activity guidelines (≥5 days per week)*												
Low SEP	450	32.8 (28.1, 37.6)	300	34.8 (29.0, 40.7)	417	32.2 (27.2, 37.2)	463	35.6 (30.7, 40.4)	2.0 (−5.1, 9.1)	3.4 (−3.0, 9.8)	−1.4 (−10.9, 8.1)	22.5 (5.1, 39.9)^d^
High SEP	144	29.4 (21.7, 37.2)	287	51.1 (44.0, 58.3)	127	47.0 (37.7, 56.2)	368	47.5 (40.7, 54.4)	21.7 (11.5, 31.8)^b^	0.6 (−11.0, 12.1)	21.1 (6.5, 35.7)^c^	
*Meets sedentary guidelines (≥5 days per week)*												
Low SEP	429	81.9 (78.2, 85.5)	299	73.9 (67.0, 78.9)	413	78.6 (74.7, 82.5)	462	74.9 (71.1, 78.8)	−7.9 (−14.1, − 1.8)^b^	−3.7 (−9.3, 2.0)	−4.3 (− 12.6, 4.1)	9.0 (−4.6, 22.7)
High SEP	135	80.2 (73.2, 87.0)	285	83.5 (78.7, 88.3)	126	84.3 (78.1, 90.5)	367	83.0 (78.5, 87.4)	3.4 (−5.0, 11.8)	−1.4 (−9.4, 6.7)	4.7 (−6.2, 15.7)	
*Active transport to or from school*												
Low SEP	449	27.4 (20.0, 34.9)	300	28.1 (19.7, 36.6)	417	32.5 (24.2, 40.9)	463	27.2 (19.7, 34.8)	0.7 (−6.2, 7.6)	−5.3 (−11.5, 0.9)	6.0 (−3.3, 15.3)	−20.8 (− 38.6, − 3.0)^d^
High SEP	144	30.9 (20.6, 41.3)	287	24.9 (15.9, 33.9)	127	26.5 (15.5, 37.5)	368	35.3 (24.6, 45.9)	−6.0 (−16.9, 4.9)	8.8 (−2.0, 19.5)	−14.8 (−3.0, 0.3)	
*Meets vegetable guidelines*												
Low SEP	445	16.5 (13.0, 20.0)	299	13.3 (9.4, 17.1)	415	19.1 (15.3, 22.9)	461	17.2 (13.8, 20.7)	−3.3 (−8.4, 1.9)	−1.9 (−7.0, 3.3)	−1.3 (− 8.7, 6.0)	−4.0 (− 17.0, 8.9)
High SEP	143	16.6 (10.4, 22.7)	286	17.4 (12.5, 22.4)	126	15.3 (9.0, 21.6)	363	21.5 (16.3, 26.7)	0.8 (−6.7, 8.4)	6.2 (−2.3, 14.8)	−5.4 (−16.1, 5.4)	
*Meets fruit guidelines*												
Low SEP	440	69.6 (65.1, 74.1)	301	71.5 (66.3, 76.7)	413	71.9 (67.4, 76.4)	463	77.6 (73.8, 81.5)	1.9 (−4.7, 8.6)	5.7 (0, 11.5)	−3.8 (−12.6, 5.0)	1.8 (− 12.8, 16.5)
High SEP	140	75.3 (68.0, 82.7)	287	73.9 (67.9, 79.9)	125	83.0 (76.4, 89.5)^a^	368	83.6 (79.0, 88.1)	−1.4 (−10.7, 7.9)	0.6 (−7.7, 8.8)	−2.0 (− 13.7, 9.7)	
*Takeaway (≤1 per week)*												
Low SEP	450	87.4 (84, 90.8)	301	86.4 (82.2, 90.6)	418	88.3 (84.8, 91.8)	462	84.3 (80.6, 88.0)	−1.0 (−6.0, 3.9)	−4.0 (−8.5, 0.6)	2.9 (−3.8, 9.6)	5.6 (− 4.1, 15.3)
High SEP	143	91.1 (86.2, 96.0)	287	93.9 (90.6, 97.1)	127	97.0 (94.1, 100)	368	91.2 (87.5, 94.9)	2.7 (−3.0, 8.5)	−5.8 (−10.5, − 1.1)^b^	8.5 (1.5, 15.6)^c^	
*Packaged snacks (≤1 per day)*												
Low SEP	441	67.0 (62.6, 71.3)	301	68.5 (63.4, 73.7)	406	71.0 (66.7, 75.2)	463	68.7 (64.7, 72.6)	1.5 (−5.2, 8.1)	−2.3 (−8.6, 3.9)	3.9 (− 5.4, 13.0)	7.4 (−6.6, 21.5)
High SEP	141	75.0 (67.7, 82.5)	287	82.8 (78.1, 87.5)	124	87.5 (81.8, 93.2)^a^	368	83.9 (79.8, 88.0)	7.5 (−1.0, 15.9)	−3.6 (− 11.1, 4.0)	11.3 (0.5, 22.0)^c^	
*Water (≥5 glasses per day)*												
Low SEP	407	52.9 (47.7, 58.1)	303	45.9 (39.9, 51.9)	374	58.2 (52.8, 63.6)	463	48.3 (42.6, 53.2)	−7.0 (−14.5, 0.5)	−9.9 (− 16.7, −3.1)^b^	2.9 (− 7.3, 13.0)	3.0 (− 15.2, 21.2)
High SEP	131	50.1 (41.2, 59.0)	287	60.0 (53.1, 66.8)	112	52.2 (42.6, 61.8)	368	56.2 (49.6, 62.7)	9.8 (−0.9, 20.6)	4.0 (−7.8, 15.7)	5.9 (−9.3, 21.1)	
*Sugar Sweetened Beverage (≤1 per day)*												
Low SEP	449	76.8 (72.5, 81.1)	301	80.9 (76.2, 85.6)	415	79.3 (75.0, 83.7)	463	85.0 (81.5, 88.5)	4.1 (−1.6, 9.9)	5.7 (0.7, 10.6)^b^	−1.5 (−9.1, 6.0)	−4.4 (− 16.0, 7.1)
High SEP	144	92.1 (87.6, 96.7)^a^	287	86.9 (82.0, 91.9)	127	90.6 (85.5, 95.6)^a^	366	91.3 (87.6, 95.0)	−5.2 (−11.8, 1.4)	0.7 (−5.4, 6.9)	−6.0 (− 14.7, 2.8)	
**Health-Related Quality of Life** (mean scores)												
*PedsQL psychosocial score*												
Low SEP	436	75.5 (73.8, 77.3)	301	73.4 (71.3, 75.5)	412	78.1 (76.2, 80.0)	462	73.9 (72.1, 75.6)	−2.2 (−4.5, 0.2)	−4.2 (−6.3, −2.1)^b^	2.1 (−1.1, 5.2)	3.1 (− 2.6, 8.8)
High SEP	140	76.8 (74.0, 79.6)	286	75.9 (73.5, 78.3)	125	81.7 (78.7, 84.8)	369	75.6 (73.3, 77.9)	−0.9 (−4.3, 2.5)	−6.1 (−9.7, −2.5)^b^	5.2 (0.4, 10.0)^c^	
*PedsQL physical score*												
Low SEP	436	82.0 (80.4, 83.6)	300	80.6 (78.7, 82.5)	412	85.5 (83.9, 87.2)	461	82.1 (80.5, 83.6)	−1.4 (−3.7, 0.9)	−3.4 (−5.5, − 1.4)^b^	2.0 (− 1.0, 5.1)	2.1 (− 3.4, 7.6)
High SEP	140	84.6 (82.0, 87.3)	286	85.1 (82.9, 87.3)	125	87.7 (84.9, 90.6)	369	84.1 (82.0, 86.1)	0.5 (−2.8, 3.7)	−3.7 (−7.2, −0.2)^b^	4.1 (−0.5, 8.7)	
*PedsQL global score*												
Low SEP	438	77.7 (76.1, 79.2)	301	75.9 (74.0, 77.8)	411	80.7 (79.0, 82.3)	462	76.8 (75.2, 78.3)	−1.8 (−3.9, 0.3)	− 3.9 (−5.8, −2.0)^b^	2.1 (−0.7, 4.9)	2.8 (− 2.4, 7.9)
High SEP	140	79.5 (76.9, 82.0)	287	79.1 (76.9, 81.1)	125	83.9 (81.1, 86.6)	369	78.6 (76.5, 80.7)	−0.4 (−3.5, 2.7)	−5.3 (−8.5, −2.0)^b^	4.9 (0.6, 9.2)^c^	

Estimates and 95% CI from linear mixed models and generalised estimating equations. Models included timepoint, group, SEP, two and three-way interactions between timepoint, group and SEP and school type, gender and age

^a^ p < 0.05 for difference between Low and High SEP within study group at 2015; ^b^ *p* < 0.05 for change between 2015 and 2019 within study group and SEP strata; ^c^ p < 0.05 for intervention effect (difference in change between intervention and control), within SEP strata; ^d^ p < 0.05 for effect modification, i.e. difference in intervention effect between SEP strata

SEP: socioeconomic position; BMI: body mass index; PedsQL: Paediatric Quality of Life

### Intervention effects

#### Weight outcomes

There was no intervention effect for weight outcomes (BMI z-score or prevalence overweight/obese) for high or low SEP students between 2015 and 2019 (Table 2 and supplementary Fig. 1a).

#### Weight-related behaviours

SEP modified the effect of the intervention on the prevalence of students meeting PA guidelines, with no effect of the intervention in students attending low SEP schools (− 1.4, 95%CI -10.9, 8.1) and a positive effect on students attending high SEP schools (+ 21.1; 95% CI 6.5, 35.7). The difference in effect between students attending high and low SEP schools was 22.5 (95%CI 5.1, 39.9) percentage points (last column, Table 2). Further analysis (Supplementary Table 1) show that the PA results are primarily attributable to girls. The difference in effect between girls attending high and low SEP schools was 30.4 (95% CI 7.2, 53.5) percentage points, primarily driven by a 19.8 (95%CI 0.4, 39.3) increase in girls from high SEP schools in intervention communities.

The intervention had a different effect on the prevalence of student using active transport to or from school for the two SEP strata. The effect was positive for low SEP students and negative for high SEP students. Although within strata the intervention effect was not significant, the difference in intervention effect between SEP groups was significant, with a − 20.8 (95%CI -38.6, − 3.0) percentage point difference.

There was a significant intervention effect on the prevalence of consuming one or fewer takeaway meals per week for high SEP students (8.5%; (95% CI 1.50, 15.6)). This was primarily driven by a decreased prevalence of students who consumed one or fewer takeaway meals per week (therefore increased consumption) in control communities (− 5.8 (95% CI -10.5, − 1.1). For low SEP students, there was no significant intervention effect for takeaway food consumption. The difference in the intervention effect on takeaway consumption between low and high SEP children was not significant.

Similarly, there was a significant intervention effect on the prevalence of consuming one or fewer packaged snacks per day for high SEP students (11.3% (95% CI 0.5, 22.0)), driven by reduced consumption in intervention students and increased consumption in control students (both non-significant within group). There was no intervention effect in low SEP students and no significant effect modification by SEP.

There was no intervention effect for high or low SEP students, or effect modification for meeting sedentary behaviour guidelines, meeting fruit or vegetable guidelines, water and SSB consumption (Supplementary Fig. 1b).

#### Health-related quality of life

There was a significant intervention effect for high SEP students for the PedsQL psychosocial score (5.2 (95% 0.4, 10.0)) and global score (4.9 (95%CI 0.6, 9.2)). Both outcomes were a result of a reduction in scores for control students and maintenance of scores in the intervention students. In low SEP schools, the intervention showed positive but not significant effects on PedsQL scores, with no significant effect modification for any of the PedsQL domains. Additional analysis (Supplementary Table 1) showed significant effect modification for the PedsQL global score for boys (7.1 (95% CI 0.1, 14.1)). This was primarily due to a 5.6 point (95% CI -9.8, − 1.3) reduction in the score for high SEP boys in control communities.

#### Gender differences

The stratified analysis by SEP and gender did not show significant differences between boys and girls in the pattern of effect modification by SEP (Supplementary Table 1).

## Discussion

This study analysed the results of a large systems-based CBI conducted in a regional area of Australia according to school-level SEP. Although not adequately powered to detect SEP differences, there were a number of significant findings in this hypothesis generating study. Overall, we did not observe any impact of the intervention on weight-related outcomes (BMI-z or proportion overweight/obesity), and this result did not vary for children attending schools classified as high or low SEP. The intervention had positive effects on PA levels among high SEP students only. HRQoL outcomes declined in control communities, while there was no change in intervention communities, with no difference in outcomes between high and low SEP students. To our knowledge, this is the first study to conduct an in-depth analysis of weight, weight-related behaviours and HRQoL outcomes of a systems-based CBI approach to childhood obesity according to SEP.

The WHO STOPS intervention did not result in a significant impact on weight outcomes in the overall 2015–2019 timeframe, and this result did not vary by school SEP. This result is in contrast to other CBIs [21, 22]. An Australian based CBI study (Be Active Eat Well) [22], and a US based CBI (Shape Up Somerville) [21] both focussed on a single community and found positive overall intervention effects on BMI-z. Interventions had a greater impact for low compared to high SEP students in the Be Active Eat Well study, while there was no difference according to SEP in Shape Up Somerville.

There are a number of possible reasons for the differing outcomes between these two studies and the WHO STOPS results. In regards to the overall effectiveness, both Be Active Eat Well [22] and Shape Up Somerville [21] focussed on a single community, whereas WHO STOPS included five intervention communities across a large geographical area, with the intervention actions, and degree of engagement varying between communities. Additionally, WHO STOPS was evaluated over four years, while Be Active Eat Well [22] and Shape Up Somerville [21] were conducted over one and two years respectively. Different SEP measures were also used. Be Active Eat Well included individual and area-level SEP measures, and Shape Up Somerville included individual measures, while WHO STOPS used a school-level SEP measure.

Additional analysis of the interventions within CBIs may also assist in identifying elements that could drive differential outcomes by SEP. Intervention strategies can be considered on a spectrum from agentic to structural, with varying implications for health equity [38]. Agentic interventions can be classified as those that target individual (or agent) behaviour change, while structural interventions are aimed at changing the policy, social, fiscal and physical environments in which unhealthy behaviours occur [38]. Evidence indicates that agentic interventions may exacerbate inequalities in health, as those with greater social and economic resources have more opportunities and ability to follow health advice [39]. At the other end of the spectrum, structural interventions, are more likely to reduce health inequalities, particularly when delivered at a community level [40–42] because agentic barriers to access, like cost, education and income level are not determining outcomes. While difficult to quantify, differing ratios of structural, agentic and agento-structural actions within the intervention communities may contribute to varying outcomes of CBI projects.

Historically, CBI studies aimed at childhood obesity have not routinely reported on behavioural outcomes according to SEP, despite including such behavioural components in their interventions [21, 22, 43, 44]. This highlights a potential gap in our understanding of how these interventions may impact differentially on weight-related behaviours, and thus impact weight status, according to SEP. Evaluating only weight outcomes by SEP may miss positive interim shorter term changes, such as improved dietary or PA behaviours, which may contribute to longer term improvement in weight status, as well as achieving other benefits associated with healthier behaviours.

The PA results of this study showed high SEP students benefitting to a greater extent from the intervention compared to low SEP students. This result may be primarily driven by girls, although this result should be interpreted with caution given the small sample once multiple stratifications were applied. The overall PA results may be due to a variety of factors. While the study intentionally took a community wide approach, there was not a particular emphasis on engaging lower SEP community members in the process of designing and/or implementing actions. Therefore, lower SEP perspectives may not have been adequately represented in the design and implementation of the actions, leading to more limited impact on low SEP students. The WHO STOPS intervention actions included community running groups, drop-off zones around schools, walking school buses and bike safety lessons. Such interventions require relatively high levels of agency and in some cases financial resources (e.g. bike safety lessons), that may favour children from higher SEP backgrounds. The one other CBI that did analyse PA outcomes by SEP, the Healthy Living Cambridge Kids study in the US, included a greater degree of structural change aimed at increasing PA levels, including school-based interventions such as increasing compulsory school PA. That study found equal improvements in fitness levels across SEP groups [18].

The dietary results show that those students attending high SEP schools had healthier dietary behaviours overall in regards to fruit, SSB and takeaway consumption at baseline. Over the intervention period, in high SEP students, takeaway and packaged snack consumption increased in control communities while declining in intervention communities. However, the intervention effect was not significantly different between high and low SEP students. Other CBI studies that have presented SEP results have not included takeaway or packaged snack consumption outcomes, however an Australian school-based obesity prevention program found no changes in takeaway consumption over 3 years in control or intervention schools [45]. Australian studies using area-level [46] and individual-level [47] SEP measures show greater takeaway consumption for low compared to high SEP children. There are a number of potential reasons for our results. The food retail environment in these communities is unknown, with any changes over this time (e.g. opening of new takeaway outlets in control communities) potentially impacting behaviours [48, 49]. Like the PA results, intervention efforts may have inadvertently been more beneficial to high SEP students.

The overall HRQoL results show students in the intervention communities maintained their wellbeing, while there were significant declines in control communities across both SEP strata. While the magnitude of the intervention effect is considered clinically significant (> 4.5 points) [36], we did not observe a significant difference in intervention effect between high and low SEP students for any of the three HRQoL domains. However, stratification by gender did show a difference in intervention effect for the global score in boys, primarily due to a reduction in scores in boys attending high SEP schools in control communities. Few other obesity focussed CBIs have considered HRQoL outcomes, and those that have [45], did not present findings according to SEP. A meta-analysis including 11 school-level intervention studies focussing on the impact of PA interventions found no overall change in HRQoL outcomes [50], however, these were also not presented according to SEP measures.

There are a number of strengths of this study. These include being one of the first cluster-randomised trials taking a systems approach to obesity prevention, as has been recommended by the latest Cochrane Review on childhood obesity intervention studies [16]. The relatively long four-year follow-up period and the use of objective anthropometric measures taken by trained researchers and health professionals, add strength to the study. The use of a passive, opt-out recruitment approach resulted in high participation rates and is important for the SEP analysis given the known association between SEP and non-participation bias in studies using active consent processes [51, 52]. A further strength of this study is analysis of all outcomes by SEP, with this element being absent in a majority of childhood obesity CBI studies.

This study also had a number of limitations. The main limitation is that it was not adequately powered, with sufficient sample sizes, to detect differences between SEP strata. Therefore, we cannot know if a lack of intervention effect within SEP strata represents a true null effect of the intervention or an inability to detect significant impacts. Nevertheless, the importance of analyses of sub-group differences, which are rarely adequately powered, is outlined by Petticrew et al. [53]. These authors suggest ensuring such sub-group analyses are theoretically sound, based on previous studies and include interaction tests, both aspects incorporated into this current study. Further, Sun et al. [54] recommend consideration of sub-group results from a ‘plausibility’ perspective. The results from this study would be considered plausible.

Using a repeat cross-sectional study design, rather than a longitudinal approach, may be considered a further limitation. However, the design of the WHO STOPS study was based on a monitoring strategy, whereby all schools in the participating LGAs were invited to take part at each wave [19]. As a result, not all the same schools participated in the 2015 and 2019 data collection waves, limiting the opportunity for longitudinal analysis. Additionally, as only year 2, 4 and 6 students participated in the data collection, and year 2 students did not complete the behavioural questionnaires, longitudinal change in the behavioural and HRQoL outcomes between 2015 and 2019 would not have been available.

SEP analysis at the school level does not allow for exploration of individual SEP factors, which have been shown to be associated with SEP gradients of childhood obesity [2, 55], nor does it allow for investigation of socio-economic variation within schools. However, with the school-based data collection, acquiring this level of information from children (e.g. parental education, household income) would not be feasible. Another commonly used measure of SEP in Australia is an area-level measure based on residential postcode data [56], however this indicator is insufficient in this regional community. As an example, one participating regional town is covered by a single postcode, however, six schools participated. When using ICSEA, these six schools were broken down into two high SEP and four low SEP schools, giving greater granularity than would be possible using a postcode-level measure. Given the community level of intervention, measuring the SEP of the school, which takes into consideration both student and school level factors, may provide a more complete picture of the environment that the students are exposed to. We may have also lost some power by using a dichotomous SEP indicator (rather than a continuous measure), however, our approach allows for ease of understanding and interpretation, particularly in regards to the development of public health recommendations.

Due to the nature of cluster-randomised control trials, and the bordering of control and intervention communities, we cannot know the potential for intervention effects to spread into neighbouring communities. Given the large geographical spread of communities in this study region, diffusion of the interventions into the control areas may be less of an issue than would be expected in urban areas. The use of self-report measures of PA and dietary behaviours included in this study may be subject to recall bias [57] and this bias could be systematically different between students attending high and low SEP schools. We have aimed to reduce this risk by using questionnaires that have been shown to be reliable in children of this age [31, 33], however future research with objectively measured PA, using for example accelerometry, would add strength to such research.

## Conclusion

These results indicate that the intervention did have equal impacts on HRQoL outcomes, however the impacts on several weight-related behaviours varied by SEP. These findings support the need to develop CBIs with an explicit equity lens by including a greater degree of structural change and ensuring inclusion of lower SEP perspectives in planning and implementation of interventions. Analysis of weight-related behaviours and HRQoL, as well as weight outcomes from an equity perspective, with larger sample sizes, is warranted to assess the impacts of CBI studies across socio-economic groups. This will contribute to the current knowledge on the most equitable way to prevent childhood obesity.

## Supplementary Information

**Additional file 1: Supplementary Fig. 1a.** Changes in predictive margins from 2015 to 2019 for weight and HRQoL outcomes. Graphical representation of changes in weight and HRQoL outcomes by intervention group and SEP

**Additional file 2: Supplementary Fig. 1b.** Changes in predictive margins from 2015 to 2019 for weight-related behaviours outcomes. Graphical representation of changes in weight-related behavioural outcomes by intervention group and SEP

**Additional file 3: Supplementary Table 1.** Anthropometric, weight-related behavioural and health-related quality of life outcomes, stratified by SEP and gender. Results table with stratification by SEP and gender and four-way interaction

## Data Availability

The datasets used and/or analysed during the current study are available from the corresponding author on reasonable request.

## Abbreviations

AT: Active transport
BAEW: Be Active Eat Well
BMI: Body Mass Index
CBI: Community Based Intervention
HRQoL: Health Related Quality of Life
PedsQL: Paediatric Quality of Life
ICSEA: Index of Community Socio-Educational Advantage
IRSAD: Index of Relative Socio-Economic Advantage and Disadvantage
MVPA: Moderate to vigorous physical activity
PA: Physical activity
SEP: Socio-Economic Position
SSB: Sugar-sweetened Beverages
WHO: World Health Organisation
WHO STOPS: Whole of Systems Trial of Prevention Strategies for Childhood Obesity
