# Supplementary figures and images for: The impact of a community-based intervention on weight, weight-related behaviours and health-related quality of life in primary school children in Victoria, Australia, according to socio-economic position
Source: BMC Public Health. 2021 Nov 27;21:2179. doi: 10.1186/s12889-021-12150-4 (PMC8627608; doi:10.1186/s12889-021-12150-4)

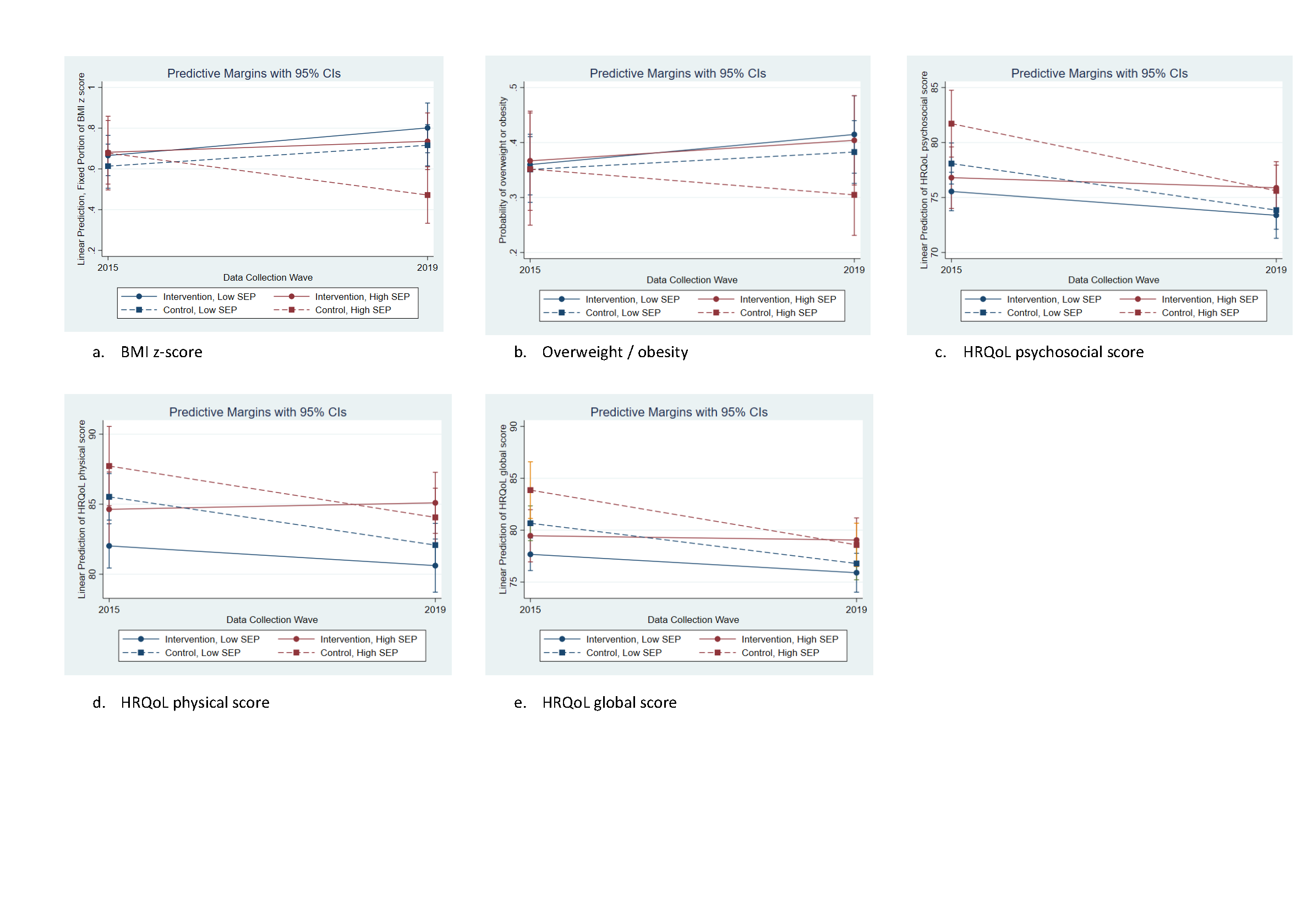

Supplement: Supplementary file 1 — Additional file 1: Supplementary Fig. 1a. Changes in predictive margins from 2015 to 2019 for weight and HRQoL outcomes. Graphical representation of changes in weight and HRQoL outcomes by intervention group and SEP [file 12889_2021_12150_MOESM1_ESM.png]

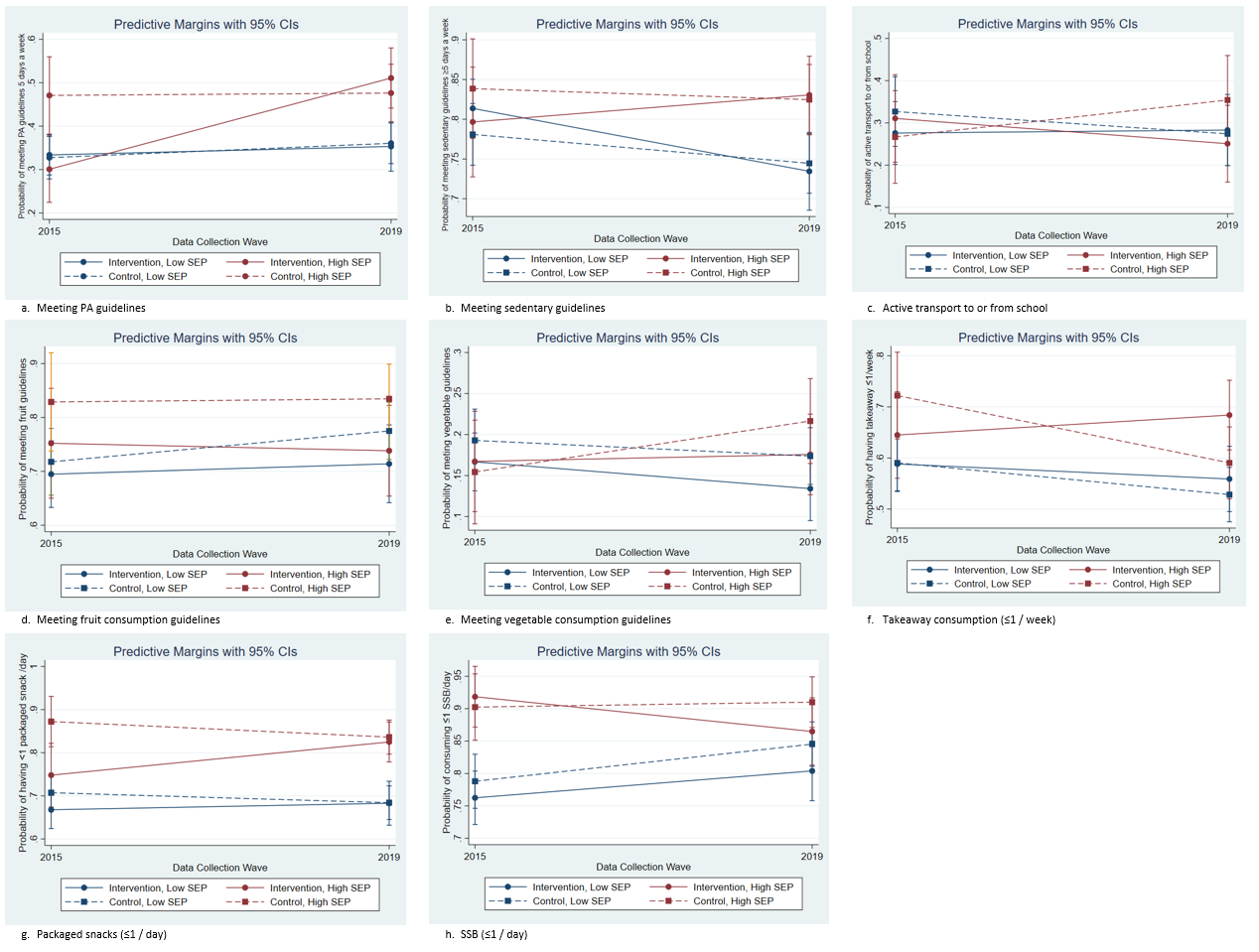

Supplement: Supplementary file 2 — Additional file 2: Supplementary Fig. 1b. Changes in predictive margins from 2015 to 2019 for weight-related behaviours outcomes. Graphical representation of changes in weight-related behavioural outcomes by intervention group and SEP [file 12889_2021_12150_MOESM2_ESM.png]
