# Supplementary material for: The impact of a community-based intervention on weight, weight-related behaviours and health-related quality of life in primary school children in Victoria, Australia, according to socio-economic position
Source: BMC Public Health. 2021 Nov 27;21:2179. doi: 10.1186/s12889-021-12150-4 (PMC8627608; doi:10.1186/s12889-021-12150-4)
Supplement: Supplementary file 3 — Additional file 3: Supplementary Table 1. Anthropometric, weight-related behavioural and health-related quality of life outcomes, stratified by SEP and gender. Results table with stratification by SEP and gender and four-way interaction [file 12889_2021_12150_MOESM3_ESM.docx]

|  | Intervention | | | | Control | | | | Change 2019 - 2015 | | Intervention effect (difference in change) | Effect modification by SEP (difference in intervention effect between SEP levels) | p value for overall four- way interaction effect |
| --- | --- | --- | --- | --- | --- | --- | --- | --- | --- | --- | --- | --- | --- |
|  | 2015 | | 2019 | | 2015 | | 2019 | | Intervention | Control | Int - Control | High - low SEP |  |
|  | n | Estimate(95% CI) | n | Estimate(95% CI) | n | Estimate(95% CI) | n | Estimate(95% CI) | Estimate(95% CI) | Estimate(95% CI) | Estimate(95% CI) | Estimate(95% CI) |  |
| **Anthropometry** | | | | | | | | | | | | |  |
| *BMI z score (mean) - boys* | | | | | | | | | | | | |  |
| Low SEP | 372 | 0.60 (0.48, 0.73) | 245 | 0.85 (0.70, 1.00) | 310 | 0.64 (0.50, 0.78) | 376 | 0.72 (0.59, 0.85) | 0.25 (0.64, 0.43) ^b^ | 7.8 (-0.09, 0.25) | 0.17 (-0.08, 0.42) | 0.07 (-0.39, 0.52) | p=0.32 |
| High SEP | 124 | 0.65 (0.44, 0.86) | 207 | 0.67 (0.49, 0.84) | 100 | 0.64 (0.40, 0.87) | 281 | 0.41 (0.25, 0.58) | 0.01 (-0.25, 0.28) | -0.22 (-0.50, 0.05) | 0.24 (-0.14, 0.61) |  |  |
| *BMI z score (mean) - girls* | | | | | | | | | | | | |  |
| Low SEP | 352 | 0.73 (0.60, 0.86) | 207 | 0.74 (0.57, 0.91) | 320 | 0.59 (0.45, 0.71) | 316 | 0.71 (0.57, 0.85) | 0.01 (-0.19, 0.20) | 0.12 (-0.06, 0.30) | -0.11 (-0.38, 0.15) | 0.39 (-0.08, 0.87) |  |
| High SEP | 118 | 0.71 (0.50, 0.93) | 209 | 0.81 (0.63, 0.98) | 85 | 0.73 (0.47, 0.98) | 255 | 0.54 (0.37, 0.72) | 0.09 (-0.17, 0.36) | -0.18 (-0.48, 0.11) | 0.29 (-0.11, 0.67) |  |  |
| O*verweight or obese (prevalence)* - *boys* | | | | | | | | | | | | |  |
| Low SEP | 372 | 34.1 (29.9, 39.3) | 245 | 42.3 (35.7, 48.9) | 310 | 34.4 (28.8, 40.2) | 376 | 39.3 (33.9, 44.6) | 8.1 (0.2, 16.1) | 4.8 (-2.4, 12.0) | 3.4 (-7.4, 14.1) | 6.4 (-12.5, 25.3) | p = 0.92 |
| High SEP | 124 | 35.4 (26.7, 44.1) | 207 | 39.3 (31.7, 46.9) | 100 | 32.9 (23.4, 42.4) | 281 | 27.1 (20.9, 33.2) | 3.9 (-7.3, 15.2) | -5.8 (-17.0, 5.3) | 9.8 (-5.8, 25.3) |  |  |
| O*verweight or obese (prevalence)* - *girls* | | | | | | | | | | | | |  |
| Low SEP | 352 | 37.8 (32.4, 43.3) | 207 | 40.3 (33.2, 47.3) | 320 | 35.7 (30.0, 41.3) | 316 | 36.9 (31.3, 42.6) | 2.5 (-6.0, 10.9) | 1.2 (-6.3, 8.7) | 1.2 (-10.1, 12.5) | 5.7 (-14.4, 25.8) |  |
| High SEP | 118 | 37.9 (28.9, 47.0) | 209 | 41.6 (34.1, 49.0) | 85 | 37.7 (27.1, 48.4) | 255 | 34.4 (27.3, 41.5) | 3.6 (-7.8, 15.1) | -3.3 (-15.9, 9.3) | 7.0 (-9.7, 23.6) |  |  |
| **Weight-related behaviours** (prevalence) | | | | | | | | | | | | |  |
| *Meets physical activity guidelines (≥5 days per week) – boys* | | | | | | | | | | | | |  |
| Low SEP | 228 | 39.8 (33.1, 46.4) | 163 | 43.0 (35.0, 50.9) | 191 | 43.6 (36.1, 51.0) | 256 | 39.3 (33.0, 45.6) | 3.2 (-6.8, 13.2) | -4.3 (-13.5, 5.0) | 7.5 (-6.2, 21.1) | 14.2 (-10.1, 38.4) | p = 0.22 |
| High SEP | 75 | 38.4 (27.1, 49.7) | 142 | 61.2 (52.2, 70.3) | 66 | 53.5 (41.2, 66.0) | 180 | 54.7 (46.4, 63.1) | 22.8 (8.7, 36.9) ^b^ | 1.2 (-13.8, 16.1) | 21.6* (1.6, 41.7) ^c^ |  |  |
| *Meets physical activity guidelines (≥5 days per week) – girls* | | | | | | | | | | | | |  |
| Low SEP | 222 | 26.3 (20.3, 32.4) | 135 | 26.9 (19.2, 34.6) | 226 | 21.9 (16.3, 27.6) | 202 | 33.2 (26.5, 39.9) | 0.6 (-8.9, 10.1) | 11.3 (2.8, 19.7) ^b^ | -10.7 (-23.3, 2.0) | 30.4 (7.2, 53.5) ^d^ |  |
| High SEP | 69 | 21.0 (11.3, 30.6) | 144 | 40.7 (31.8, 49.7) | 61 | 40.2 (27.6, 52.9) | 186 | 40.2 (31.7, 48.6) | 19.7 (6.8, 32.6) ^b^ | 1.2 (-13.8, 16.1) | 19.8 (0.4, 39.3) ^c^ |  |  |
|  |  |  |  |  |  |  |  |  |  |  |  |  |  |
|  | Intervention | | | | Control | | | | Change 2019 - 2015 | | Intervention effect (difference in change) | Effect modification by SEP (difference in intervention effect between SEP levels) | p value for overall four- way interaction effect |
|  | 2015 | | 2019 | | 2015 | | 2019 | | Intervention | Control | Int - Control | High - low SEP |  |
|  | n | Estimate(95% CI) | n | Estimate(95% CI) | n | Estimate(95% CI) | n | Estimate(95% CI) | Estimate(95% CI) | Estimate(95% CI) | Estimate(95% CI) | Estimate(95% CI) |  |
| *Meets sedentary guidelines (≥5 days per week) - boys* | | | | | | | | | | | | | |
| Low SEP | 213 | 77.6 (73.0, 83.1) | 162 | 66.9 (59.7, 74.1) | 189 | 71.3 (65.0, 77.6) | 255 | 68.4 (62.8, 73.9) | -10.7 (-19.8, -1.6) ^b^ | -2.9 (-11.6, 5.7) | -7.7 (-10.3, 4.8) | 18.8 (-2.0, 39.6) | p = 0.25 |
| High SEP | 69 | 72.7 (62.0, 83.4) | 142 | 83.7 (77.1, 90.2) | 65 | 79.8 (70.2, 89.4) | 180 | 79.7 (73.5, 86.0) | 11.0 (-1.6, 23.5) | -0.1 (-11.9, 11.8) | 11.0 (-5.6, 27.7) |  |  |
| *Meets sedentary guidelines (≥5 days per week) - girls* | | | | | | | | | | | | |  |
| Low SEP | 216 | 85.5 (80.8, 90.1) | 135 | 80.5 (73.9, 87.1) | 224 | 84.7 (80.1, 89.4) | 202 | 80.9 (75.6, 86.2) | -5.0 (-13.2, 3.2) | -3.9 (-11.0, 3.3) | -1.1 (-12.0, 9.7) | -0.5 (-18.2, 17.1) |  |
| High SEP | 66 | 87.3 (79.0, 95.5) | 142 | 82.9 (76.3, 89.5) | 61 | 88.2 (80.2, 96.2) | 186 | 85.5 (80.2, 90.9) | -4.4 (-14.9, 6.2) | -2.7 (-12.6, 7.3) | -1.7 (-15.7, 12.3) |  |  |
| *Active transport to or from school - boys* | | | | | | | | | | | | | |
| Low SEP | 226 | 29.5 (20.8, 38.1) | 163 | 34.7 (24.5, 44.9) | 190 | 29.7 (20.2, 39.1) | 256 | 28.8 (20.3, 37.4) | 5.2 (-4.4, 14.9) | -0.8 (-9.3, 7.7) | 6.0 (-6.8, 18.9) | -16.5 (-40.5, 7.5) | p = 0.62 |
| High SEP | 75 | 32.1 (19.3, 44.8) | 142 | 26.2 (15.6, 36.8) | 66 | 33.4 (19.5, 47.3) | 180 | 38.1 (26.4, 49.9) | -5.8 (-20.1, 8.5) | 4.7 (-9.8, 19.2) | -10.5 (-30.6, 9.6) |  |  |
| *Active transport to or from school - girls* | | | | | | | | | | | | |  |
| Low SEP | 223 | 25.0 (16.7, 33.3) | 135 | 20.3 (11.3, 29.3) | 227 | 34.9 (25.5, 44.2) | 202 | 26.2 (17.5, 34.8) | -4.7 (-13.7, 4.2) | -8.7 (-17.3, -0.1) ^b^ | 4.0 (-8.4, 16.3) | -23.1 (-0.46, -0.4) ^d^ |  |
| High SEP | 69 | 30.1 (17.1, 43.0) | 144 | 23.6 (13.5, 33.6) | 61 | 18.8 (6.5, 31.0) | 186 | 31.4 (20.0, 42.9) | -6.5 (-20.7, 7.6) | 12.6 (-0.3, 25.6) | -19.2 (-0.38, -0.2) ^c^ |  |  |
| *Meets vegetable guidelines - boys* | | | | | | | | | | | | | |
| Low SEP | 223 | 12.3 (8.0, 16.7) | 162 | 11.8 (6.8, 16.8) | 189 | 20.1 (14.4, 25.9) | 254 | 17.5 (12.8, 22.2) | -0.5 (-7.2, 6.1) | -2.7 (-10.1, 4.8) | 2.1 (-7.9, 12.1) | -10.9 (-28.6, 6.8) | p = 0.33 |
| High SEP | 74 | 17.8 (9.1, 26.4) | 141 | 15.3 (9.1, 21.6) | 65 | 14.3 (5.7 (22.8) | 175 | 20.6 (14.1, 27.2) | -2.4 (-12.8, 8.0) | 6.4 (-4.7, 17.4) | -8.8 (-23.6, 6.0) |  |  |
| *Meets vegetable guidelines - girls* | | | | | | | | | | | | |  |
| Low SEP | 222 | 20.7 (15.3, 26.2) | 135 | 14.9 (8.9, 20.9) | 226 | 18.5 (13.4, 23.6) | 202 | 16.7 (11.5, 21.8) | -5.8 (-13.9, 2.2) | -1.8 (-9.1, 5.4) | -4.0 (-14.8, 6.8) | 2.2 (-16.7, 21.1) |  |
| High SEP | 69 | 15.2 (6.8, 23.7) | 144 | 19.5 (12.7, 26.3) | 61 | 16.4 (7.1, 25.7) | 186 | 22.5 (15.6, 29.3) | 4.3 (-6.3, 14.9) | 6.0 (-5.8, 17.9) | -1.8 (-17.2, 13.7) |  |  |

|  | Intervention | | | | Control | | | | Change 2019 - 2015 | | Intervention effect (difference in change) | Effect modification by SEP (difference in intervention effect between SEP levels) | p value for overall four- way interaction effect |
| --- | --- | --- | --- | --- | --- | --- | --- | --- | --- | --- | --- | --- | --- |
|  | 2015 | | 2019 | | 2015 | | 2019 | | Intervention | Control | Int - Control | High - low SEP |  |
|  | n | Estimate(95% CI) | n | Estimate(95% CI) | n | Estimate(95% CI) | n | Estimate(95% CI) | Estimate(95% CI) | Estimate(95% CI) | Estimate(95% CI) | Estimate(95% CI) |  |
| *Meets fruit guidelines - boys* | | | | | | | | | | | | | |
| Low SEP | 221 | 67.8 (61.4, 74.0) | 164 | 69.6 (62.5, 76.8) | 189 | 64.8 (57.8, 71.7) | 256 | 75.1 (68.8, 80.4) | 1.9 (-7.4, 11.2) | 10.3 (1.7, 18.9) ^b^ | -8.5 (-21.1, 4.2) | 13.5 (-7.5, 34.5) | p = 0.16 |
| High SEP | 72 | 68.9 (58.0, 79.8) | 142 | 72.6 (64.4, 80.8) | 65 | 85.1 (76.5, 93.7) | 180 | 83.8 (77.9, 89.7) | 3.7 (-9.7, 17.2) | -1.3 (-11.9, 9.2) | 5.0 (-11.6, 21.7) |  |  |
| *Meets fruit guidelines – girls* | | | | | | | | | | | | |  |
| Low SEP | 219 | 71.4 (65.3, 77.5) | 135 | 73.2 (65.7, 80.8) | 224 | 78.2 (72.8, 83.7) | 202 | 80.1 (74.6, 85.6) | 1.8 (-7.7, 11.3) | 1.9 (-5.8, 9.5) | 0 (-12.2, 12.2) | -9.2 (-29.6, 11.3) |  |
| High SEP | 68 | 82.0 (72.7, 91.3) | 144 | 75.3 (67.6, 83.1) | 60 | 80.5 (70.5, 90.5) | 186 | 83.0 (76.9, 89.2) | -6.7 (-18.6, 5.3) | 2.5 (-9.4, 14.5) | -9.2 (-25.6, 7.2) |  |  |
| *Takeaway (≤1 per week)*  - *boys* | | | | | | | | | | | | | |
| Low SEP | 227 | 84.2 (79.1, 89.2) | 164 | 79.3 (72.7, 85.9) | 191 | 83.6 (78.0, 89.3) | 255 | 78.8 (73.3, 84.2) | -4.8 (-12.7, 3.1) | -4.8 (-12.2, 2.5) | 0 (-10.8, 10.8) | 15.2 (0.3, 30.0) ^d^ | p = 0.08 |
| High SEP | 74 | 87.8 (80.0, 95.6) | 142 | 95.7 (92.1, 99.4) | 66 | 97.0 (92.8, 100.2) | 180 | 89.6 (84.5, 94.7) | 7.9 (-0.5, 16.4) | -7.4 (-13.9, -0.9) ^b^ | 15.3 (4.9, 25.8) ^c^ |  |  |
| *Takeaway (≤1 per week)*  - *girls* | | | | | | | | | | | | |  |
| Low SEP | 223 | 90.0 (85.8, 94.1) | 135 | 93.4 (89.0, 97.7) | 227 | 92.1 (88.3, 95.8) | 202 | 89.3 (84.9, 93.7) | 3.4 (-2.4, 9.1) | -2.7 (-8.2, 2.7) | 6.1 (-1.8, 14.0) | -3.7 (-16.2, 8.8) |  |
| High SEP | 69 | 94.0 (88.1, 99.9) | 144 | 91.9 (87.0, 96.8) | 61 | 96.7 (92.2, 100.3) | 186 | 92.2 (87.9, 96.6) | -2.1 (-9.6, 5.5) | -4.5 (-10.7, 1.7) | 2.4 (-7.1, 11.9) |  |  |
| *Packaged snacks (≤1 per day)*  - *boys* | | | | | | | | | | | | | |
| Low SEP | 222 | 66.5 (60.3, 72.8) | 164 | 64.1 (56.8, 71.4) | 182 | 70.8 (64.4, 77.2) | 256 | 63.4 (57.7, 69.0) | -2.4 (-12.0, 7.2) | -7.4 (-16.4, 1.6) | 5.0 (-8.2, 18.1) | 18.3 (-2.0, 38.7) | p= 0.12 |
| High SEP | 72 | 66.8 (55.6, 78.0) | 142 | 85.0 (78.9, 91.0) | 65 | 87.9 (79.9, 95.9) | 180 | 82.6 (77.1, 88.1) | 18.2 (5.5, 30.8) ^b^ | -5.3 (-15.3, 4.8) | 23.4 (7.8, 39.0) ^c^ |  |  |
| *Packaged snacks (≤1 per day)*  - *girls* | | | | | | | | | | | | |  |
| Low SEP | 219 | 67.4 (61.2, 73.7) | 135 | 73.4 (66.0, 80.9) | 224 | 71.6 (65.8, 77.4) | 202 | 74.7 (68.9, 80.5) | 6.0 (-3.8, 15.8) | 3.1 (-5.4, 11.6) | 2.9 (-10.1, 15.8) | -4.3 (-24.0, 12.3) |  |
| High SEP | 69 | 83.9 (74.8 (92.9) | 144 | 80.8 (74.1, 87.4) | 59 | 86.9 (78.3, 95.5) | 186 | 85.2 (80.1, 90.3) | -3.1 (-14.3, 8.1) | -1.7 (-12.0, 8.7) | -1.4 (-16.1, 13.3) |  |  |

|  | Intervention | | | | Control | | | | Change 2019 - 2015 | | Intervention effect (difference in change) | Effect modification by SEP (difference in intervention effect between SEP levels) | p value for overall four- way interaction effect |
| --- | --- | --- | --- | --- | --- | --- | --- | --- | --- | --- | --- | --- | --- |
|  | 2015 | | 2019 | | 2015 | | 2019 | | Intervention | Control | Int - Control | High - low SEP |  |
|  | n | Estimate(95% CI) | n | Estimate(95% CI) | n | Estimate(95% CI) | n | Estimate(95% CI) | Estimate(95% CI) | Estimate(95% CI) | Estimate(95% CI) | Estimate(95% CI) |  |
| *Water (≥5 glasses per day)* - *boys* | | | | | | | | | | | | | |
| Low SEP | 201 | 58.2 (51.1, 65.3) | 164 | 42.5 (34.7, 50.4) | 170 | 59.7 (52.0, 67.3) | 256 | 46.9 (40.5, 53.3) | -15.7 (-25.9, -5.5) ^b^ | -12.7 (-22.4, -3.1) | -3.0 (-17, 11.0) | 4.2 (-20.8, 29.2) | p= 0.90 |
| High SEP | 68 | 51.9 (39.7, 64.0) | 142 | 59.6 (50.6, 68.6) | 61 | 51.5 (38.8, 64.3) | 180 | 58.0 (49.9, 66.2) | 7.7 (-7.0, 22.5) | 6.5 (-8.7, 21.7) | 1.2 (-19.45, 21.9) |  |  |
| *Water (≥5 glasses per day)* - *girls* | | | | | | | | | | | | |  |
| Low SEP | 206 | 47.8 (40.7, 54.9) | 137 | 50.2 (41.5, 58.8) | 204 | 56.9 (49.8, 64.0) | 202 | 50.2 (43.1, 57.3) | 2.3 (-8.4, 12.2) | -6.6 (-16.4, 3.1) | 9.0 (-5.5, 23.5) | 1.8 (-24.2, 27.9) |  |
| High SEP | 63 | 48.3 (35.7, 60.9) | 144 | 60.1 (51.4, 68.9) | 51 | 53.1 (38.8, 64.3) | 186 | 54.2 (45.7, 62.6) | 11.9 (-3.2, 26.9) | 1.0 (-15.4, 17.4) | 10.8 (-10.9, 32.5) |  |  |
| *Sugar Sweetened Beverage (≤1 per day)- boys* | | | | | | | | | | | | | |
| Low SEP | 227 | 77.6 (72.0, 83.2) | 164 | 75.1 (68.3, 81.9) | 191 | 76.2 (69.9, 82.5) | 156 | 82.0 (77.1, 86.8) | -2.5 (-10.8, 5.8) | 5.8 (-1.7, 13.2) | -8.3 (-19.5, 2.8) | 3.5 (-13.6, 20.7) | p = 0.39 |
| High SEP | 75 | 89.0 (81.7, 96.3) | 142 | 89.4 (83.2, 95.5) | 66 | 86.8 (78.8, 94.8) | 180 | 92.0 (87.3, 96.6) | 0.4 (-9.1, 9.9) | 5.2 (-4.0, 14.4) | -4.8 (-17.8, 8.2) |  |  |
| *Sugar Sweetened Beverage (≤1 per day)- girls* | | | | | | | | | | | | |  |
| Low SEP | 222 | 75.9 (70.1, 81.8) | 135 | 87.4 (81.9, 92.9) | 224 | 82.4 (77.2, 87.5) | 202 | 88.3 (83.9, 92.7) | 11.5 (3.8, 19.1) ^b^ | 5.9 (-0.5, 12.4) | 5.5 (-4.4, 15.5) | -12.3 (-27.3, 2.6) |  |
| High SEP | 69 | 95.5 (90.5, 100.5) | 144 | 84.7 (77.7, 91.7) | 61 | 94.5 (89.0, 99.9) | 184 | 90.5 (85.3, 95.7) | -10.8 (-19.4, -2.2) ^b^ | -4.0 (-11.5, 3.6) | -6.8 (-18.0, 4.4) |  |  |

|  | Intervention | | | | Control | | | | Change 2019 - 2015 | | Intervention effect (difference in change) | Effect modification by SEP (difference in intervention effect between SEP levels) | p value for overall four- way interaction effect |
| --- | --- | --- | --- | --- | --- | --- | --- | --- | --- | --- | --- | --- | --- |
|  | 2015 | | 2019 | | 2015 | | 2019 | | Intervention | Control | Int - Control | High - low SEP |  |
|  | n | Estimate(95% CI) | n | Estimate(95% CI) | n | Estimate(95% CI) | n | Estimate(95% CI) | Estimate(95% CI) | Estimate(95% CI) | Estimate(95% CI) | Estimate(95% CI) |  |
| **Health-Related Quality of Life**  (mean scores) | | | | | | | | | | | | | |
| *PedsQL psychosocial score - boys* | | | | | | | | | | | | | |
| Low SEP | 222 | 75.3 (73.1, 77.6) | 162 | 72.0 (69.4, 74.7) | 186 | 76.6 (74.1, 79.0) | 255 | 73.1 (71.0, 75.3) | -3.3 (-6.5, -0.2) ^b^ | -3.4 (-6.3, -0.5) ^b^ | 0.1 (-4.2, 4.5) | 7.5 (-0.24, 15.2) | p = 0.11 |
| High SEP | 73 | 75.3 (71.6, 79.0) | 142 | 76.6 (73.7, 79.6) | 64 | 80.8 (76.9, 84.8) | 181 | 74.6 (71.8, 77.3) | 1.3 (-3.2, 5.9) | -6.3 (-11.0, -1.6) ^b^ | 7.6 (1.2, 14.0) ^c^ |  |  |
| *PedsQL psychosocial score - girls* | | | | | | | | | | | | |  |
| Low SEP | 214 | 75.8 (73.5, 78.1) | 137 | 74.8 (72.0, 77.6) | 226 | 79.5 (77.2, 81.8) | 202 | 74.5 (72.2, 76.9) | -0.9 (-4.3, 2.4) | -4.9 (-7.9, -2.0) ^b^ | 4.0 (-0.5, 8.4) | -1.2 (-9.2, 6.7) |  |
| High SEP | 67 | 78.4 (74.5, 82.2) | 143 | 75.2 (72.3, 78.2) | 61 | 82.6 (78.5, 86.8) | 186 | 76.7 (73.9, 79.6) | -3.1 (-7.8, 1.5) | -5.9 (-10.7, -1.1) ^b^ | 2.7 (-3.8, 9.3) |  |  |
| *PedsQL physical score - boys* | | | | | | | | | | | | | |
| Low SEP | 222 | 81.0 (78.9, 83.1) | 162 | 80.4 (77.9, 82.9) | 185 | 85.0 (82.7, 87.4) | 254 | 82.6 (80.6, 84.6) | -2.2 (-5.5, 1.1) | -4.5 (-7.4, -1.6) | 1.8 (-2.5, 6.1) | 5.7 (-1.9, 13.3) | p = 0.18 |
| High SEP | 73 | 83.4 (79.8, 87.0) | 142 | 86.9 (84.0, 89.7) | 64 | 88.8 (84.9, 92.6) | 181 | 84.7 (82.1, 87.3) | -2.6 (-7.2, 2.0) | -3.3 (-8.7, 0.5) | 7.5 (1.2, 13.8) ^c^ |  |  |
| *PedsQL physical score - girls* | | | | | | | | | | | | |  |
| Low SEP | 214 | 83.1 (80.9, 85.3) | 136 | 80.9 (78.2, 83.6) | 227 | 85.9 (83.8, 88.1) | 202 | 81.5 (79.2, 83.7) | -2.2 (-5.5, 1.1) | -4.5 (-7.4, -1.6) ^b^ | 2.3 (-2.1, 6.7) | -1.6 (-9.4, 6.1) |  |
| High SEP | 67 | 86.0 (82.3, 89.8) | 143 | 83.4 (80.6, 86.2) | 61 | 86.6 (82.7, 90.6) | 186 | 83.4 (80.7, 86.0) | -2.6 (-7.2, 2.0) | -3.3 (-8.0, 1.5) | 0.6 (-5.8, 7.1) |  |  |
| *PedsQL global score - boys* | | | | | | | | | | | | | |
| Low SEP | 223 | 77.2 (75.2, 79.2) | 162 | 74.9 (72.6, 77.3) | 185 | 79.5 (77.2, 81.7) | 225 | 76.5 (74.6, 78.4) | -2.2 (-5.1, 0.6) | -3.0 (-5.6, -0.3) ^b^ | 0.7 (-3.2, 4.6) | 7.1 (0.1, 14.1) ^d^ | p = 0.08 |
| High SEP | 73 | 78.0 (74.7, 81.3) | 142 | 80.2 (77.5, 82.9) | 64 | 83.7 (80.1, 87.3) | 181 | 78.1 (75.6, 80.6) | 2.2 (-1.9, 6.3) | -5.6 (-9.8, -1.3) ^b^ | 7.8 (2.0, 13.6) |  |  |
| *PedsQL global score - girls* | | | | | | | | | | | | |  |
| Low SEP | 215 | 78.2 (76.1, 80.2) | 137 | 76.9 (74.4, 79.5) | 226 | 81.7 (79.7, 83.8) | 202 | 77.0 (74.9, 79.1) | -1.2 (-4.2, 1.8) | -4.7 (-7.4, -2.0) ^b^ | 3.5 (-0.5, 7.5) | -1.6 (-8.7, 5.6) |  |
| High SEP | 67 | 81.0 (77.6, 84.5) | 144 | 78.0 (75.4, 80.6) | 61 | 84.0 (80.3, 87.8) | 186 | 79.1 (76.5, 81.7) | -3.0 (-7.2, 1.2) | -4.9 (-9.2, -0.6) ^b^ | 1.9 (-4.0, 7.8) |  |  |

Estimates and 95% CI from linear mixed models and generalised estimating equations. Models included timepoint, group, SEP, gender, two, three and four-way interactions between timepoint, group, SEP and gender, and school type and age.

^a^ p<0.05 for difference between Low and High SEP within study group at 2015; ^b^ p<0.05 for change between 2015 and 2019 within study group and SEP strata; ^c^ p<0.05 for intervention effect (difference in change between intervention and control), within SEP strata; ^d^ p<0.05 for effect modification, i.e difference in intervention effect between SEP strata;

## SEP: socioeconomic position; BMI: body mass index; PedsQL: Paediatric Quality of Life
